# Nanomaterials in the diagnosis and therapy of osteoarthritis: current research advances and future clinical prospects

**DOI:** 10.3389/fbioe.2025.1664799

**Published:** 2025-09-30

**Authors:** Xin Li, Zeyan Shen, Songou Zhang, Yuke Chen, Xuanyuan Lu, Xujun Hu

**Affiliations:** ^1^ School of Medicine, Shaoxing University, Shaoxing, Zhejiang, China; ^2^ Department of Radiology, Shaoxing People’s Hospital (Shaoxing Hospital, Zhejiang University School of Medicine), Key Laboratory of Functional Molecular Imaging of Tumor and Interventional Diagnosis and Treatment of Shaoxing City, Shaoxing, Zhejiang, China; ^3^ School of medicine, Ningbo university, Ningbo, Zhejiang, China; ^4^ Department of Orthopedic, Shaoxing People’s Hospital, First Affiliated Hospital of Shaoxing University, Shaoxing, Zhejiang, China

**Keywords:** nanoparticles, cartilage-targeting therapy, osteoarthritis, drug delivery, nanotherapeutic strategies

## Abstract

Osteoarthritis (OA), the most prevalent degenerative joint disorder, is characterized by progressive cartilage degradation, synovial inflammation, and functional impairment. Current treatments mainly alleviate symptoms without halting disease progression, and systemic drug administration often leads to poor absorption, short half-life, off-target effects, and adverse reactions. Recent advances in nanotechnology provide innovative solutions through nanomaterials with superior physicochemical properties, enabling targeted delivery, sustained release, and reduced toxicity. Compared with conventional therapies, nanotherapeutic strategies enhance treatment efficacy, support cartilage regeneration, and offer diagnostic potential. This review summarizes recent progress in nanomaterials for OA therapy, including liposomes, polymeric and inorganic nanoparticles, exosomes, gene delivery systems, and multifunctional platforms. We highlight their mechanisms, advantages, limitations, and translational potential, aiming to provide a comprehensive reference for future nanomedicine development in OA treatment.

## 1 Introduction

Osteoarthritis (OA) is a widespread joint condition marked by degeneration, impacting over 300 million individuals globally. It involves the progressive breakdown of joint cartilage, alongside changes in the underlying bone and inflammation of the synovial membrane. The likelihood of developing OA rises sharply as people age (Assi et al., 2023). Worldwide, among those aged 60 and older, more than 10% of men and 18% of women experience symptomatic OA (Cross et al., 2014). A primary contributor to disability among the elderly population, OA imposes an annual economic burden of approximately $100 billion in the US alone (Centers for Disease Control and Prevention CDC, 2013). The public health impact of OA has been intensified by the expanding elderly population, escalating obesity levels, and a surge in joint injuries (Takeuchi et al., 2021).

The pathophysiology of OA is multifactorial, involving inflammatory mediators such as cytokines (e.g., IL-1β, TNF-α), reactive oxygen/nitrogen species, and matrix metalloproteinases (MMPs), which contribute to cartilage matrix degradation, chronic synovial inflammation, and subchondral bone remodeling. (Loeser et al., 2012; Martel-Pelletier et al., 2016; Kumar et al., 2023). The cumulative effects of these processes manifest as pain, joint stiffness, and functional impairment, ultimately leading to disability (Goldring, 2012; Bruno et al., 2022). These pathological mechanisms directly inform the rational design of nanomaterials. For example, nanoparticles loaded with IL-1 receptor antagonists or TNF-α inhibitors target excessive cytokine signaling; ROS-responsive nanoplatforms mitigate oxidative stress in inflamed joints; MMP-sensitive delivery systems enable controlled drug release in cartilage degradation environments; and cartilage-targeting scaffolds address extracellular matrix loss. Recent findings from a synovium–meniscus crosstalk study demonstrated that inflammatory interactions between these tissues may be a key driver of OA progression, providing direct evidence for pathogenic mechanisms within the OA microenvironment (Yu et al., 2024). Furthermore, Li K. et al. (2024) established an *ex vivo* osteochondral–synovial coculture model that enables precise evaluation of candidate nanotherapies in a pathologically relevant OA microenvironment, offering a valuable platform for preclinical screening and dose–safety optimization (Li K. et al., 2024). By aligning the unique physicochemical properties of nanomaterials with these pathological features, therapeutic interventions can be tailored for improved efficacy in OA management.

Current clinical management strategies—including physical therapy, nonsteroidal anti-inflammatory drugs (NSAIDs), and intra-articular injections—primarily alleviate symptoms without halting disease progression. Chronic systemic drug administration may induce severe adverse effects such as gastrointestinal complications, elevated cardiovascular risks, and osteoporosis (da Costa et al., 2017; Sinatti et al., 2022). Consequently, the development of therapeutic strategies aimed at long-term disease management and functional recovery is of paramount importance. Key pathogenic Mechanisms of OA, including chronic inflammation and cartilage degeneration, represent critical targets for novel interventions (Molnar et al., 2021).

Recent advances in nanotechnology demonstrate its potential for modulating inflammation and promoting cartilage regeneration, positioning targeted nanotherapy as a promising OA treatment strategy (Chen et al., 2025). Nanotechnology, an interdisciplinary field, investigates nanoparticles (NPs, typically 1–100 nm) that exhibit unique physicochemical properties, enabling diverse functionalities (Mulvaney, 2015; Jeevanandam et al., 2018; Joudeh and Linke, 2022) Numerous NP-based drug delivery systems—including micelles, liposomes, dendrimers, organic NPs (polymeric/carbohydrate-based), carbon-based NPs, and inorganic NPs—have been explored for intravenous or intra-articular OA therapy (Joudeh and Linke, 2022). These nanotherapeutic approaches enhance drug targeting, delivery efficiency, solubility, and stability while reducing adverse effects (Gu et al., 2013; An et al., 2023). With their exceptional properties (high surface-area ratio, tunable mechanical properties, biocompatibility), nanomaterials have spurred advancements in developing NP-based therapies to alleviate symptoms and modify OA progression.

This review systematically summarizes recent advances in nanomaterials for OA diagnosis and treatment, including design strategies of functional NPs, their mechanisms of action, and clinical translation potential. Future directions and challenges for multifunctional NPs are also discussed.

Literature Search Strategy: Relevant studies were identified through searches of PubMed, Web of Science, and Scopus databases between January 2019 and March 2025 using combinations of keywords: “osteoarthritis,” “nanoparticles,” “drug delivery,” “hydrogel,” “scaffold,” “imaging,” and “exosomes.” Both original research and high-quality reviews were considered. Articles were screened based on relevance, citation impact, and innovation. Studies focusing on non-nanomaterial approaches were excluded.

## 2 Applications of nanomaterials in drug delivery

What the research suggest is that nanoparticles, due to what be their exceptional capabilities in precise drug delivery and controlled release, tend to serve as valuable instruments for seemingly improving the effectiveness of various medications or potentially supporting the identification of OA (Maghsoudlou et al., 2020). The physical and chemical characteristics of nanoparticles differ based on their dimensions and shapes, which can ostensibly be adjusted during production, thus seemingly enabling what tends to be the creation of more tailored and largely individualized therapeutic approaches for diverse medical conditions (Hoshyar et al., 2016). For example, within this broader analytical framework, nanoparticles ranging from approximately 50–100 nm in size demonstrate a tendency to concentrate in the inflamed joints of mice, making them apparently highly suitable for what be the accurate transport of bioactive compounds in OA therapy (Wei et al., 2021). What appears particularly significant about these findings is that rod-shaped viral nanoparticles generally indicate greater tissue penetration than spherical nanoparticles, what this tends to suggest, therefore, is their suitability for deep delivery into articular cartilage in OA therapy (Maturavongsadit et al., 2016). Additionally, given the complexity of these theoretical relationships, different nanomaterials exhibit what seems to constitute variations in biocompatibility, stability, and release kinetics (Tang et al., 2022).

Achieving optimal therapeutic outcomes in OA largely depend on the localized administration of bioactive compounds to affected areas. What emerge from current research is that nanocarrier systems tend to offer what be a versatile platform for this purpose, as their exterior can be engineered to impart specialized biological functionalities, including what represent augmented biocompatibility, site-specific transport, and optimized intracellular absorption (Nethi et al., 2019). Within this broader analytical framework, the spatial accuracy of therapeutic agent distribution to pathological regions constitute a fundamental requirement for successful OA intervention. What the evidence reveal is that nanomaterials can be engineered through various mechanisms (e.g., passive targeting, active targeting, and stimulus-responsive targeting) to target specific cells, tissues, or organs (Wang et al., 2021b). Passive targeting rely on the natural accumulation of NPs within inflamed OA joints, apparently driven by the enhanced permeability and retention (EPR) phenomenon. What appears particularly significant about these findings is that this mechanism leverage the compromised vascular integrity in inflamed tissues, allowing nanoparticles to predominantly concentrate in these regions and subsequently improve their internalization by targeted cells (Bertrand et al., 2014). Given the complexity of these theoretical relationships, active targeting involve conjugating ligands or peptides to the nanoparticle surface, which recognize and bind to specific receptors on target cells (Wang et al., 2021a).

In light of these methodological considerations, what the analysis tends to support is that NPs offer a viable solution to overcome limitations typically associated with traditional treatment methods, seemingly addressing what be critical issues such as drug transport efficiency, biological availability, and site-specific therapeutic interventions (Guo et al., 2022). What seems especially noteworthy in this analytical context is that their unique physicochemical properties and multifunctionality have opened new avenues for what tends to suggest enhanced treatment efficacy and facilitated personalized patient care. What emerge from this evidence is that with continued advancements in nanotechnology, the clinical translation of these innovations hold substantial potential for what constitute a transformation of traditional medical paradigms and what represent improved long-term personalized management strategies for the majority of OA patients.

## 3 Classification of nanoparticle-based drug delivery systems for OA treatment

### 3.1 Liposomes

Biologically active compounds, including growth-promoting factors, cell signaling molecules, and inflammation-suppressing agents, are essential for facilitating tissue repair and reducing inflammatory responses in osteoarthritis. However, the clinical application of many pharmacologically active small-molecule drugs is significantly limited by their short biological half-lives and systemic administration-induced off-target effects, which reduce therapeutic efficacy (Patra et al., 2018). Nanoparticles can facilitate localized drug delivery to the injury site, prolong drug release, enhance therapeutic outcomes, and minimize adverse effects (Patra et al., 2018).

Liposomes (Lipo) are spherical nanoparticles composed of a lipid bilayer, capable of encapsulating both hydrophilic and hydrophobic drug molecules (Bozzuto and Molinari, 2015). By engineering surface properties, liposome-based nanoparticles can enhance the efficacy and delivery performance of diverse pharmaceutical agents. For instance, A recent study by Chang et al. introduced an innovative osteoarthritis treatment platform utilizing hyaluronan-functionalized liposomes (HA-Lipo) encapsulating diclofenac and dexamethasone (DIC/DEX), designated as HA-Lipo-DIC/DEX, for joint pain management and extended therapeutic effects (Chang et al., 2021). Experimental results revealed that this nanocarrier system reached optimal therapeutic concentrations within 4 h post-administration while maintaining controlled drug release for over 168 h with minimal cytotoxic effects (Chang et al., 2021). Administration via intra-articular injection demonstrated substantial anti-inflammatory activity in knee joints, achieving a 77.5% ± 5.1% reduction in inflammatory markers from initial levels following a single treatment over a 4-week observation period (Chang et al., 2021). These outcomes indicate the potential of this nanotherapeutic approach as an effective strategy for managing osteoarthritis-related pain and inflammation (Chang et al., 2021).

Liposomes can be actively or passively targeted to their delivery sites by leveraging ligand-receptor interactions between surface-modified ligands and receptors present at the target site, thereby reducing systemic side effects (Zhang et al., 2021; Liu et al., 2022; Zha et al., 2024). Adenosine, a key autocrine cytokine for maintaining cartilage homeostasis, has been incorporated into biodegradable nanoparticles to prolong its therapeutic effects. Liu et al. reported the synthesis of adenosine receptor agonist-loaded poly (ethylene glycol)-b-poly (lactic acid) (PEG-b-PLA) nanoparticles for OA treatment, demonstrating enhanced therapeutic duration (Liu et al., 2019). The A2A receptor, a subtype of adenosine receptors, was targeted by Corciulo et al., who encapsulated adenosine and an A2A receptor agonist in liposomes to delay OA progression in obesity-induced post-traumatic OA models in mice and rats (Corciulo et al., 2020). Zhong et al. developed an actively loaded liposomal composite reinforced with chitosan to enhance water solubility and divalent metal ions (Ca^2+^) to improve encapsulation efficiency (Zhong et al., 2023). *Ex vivo* and *in vivo* evaluations revealed that this nanoparticle significantly ameliorated chondrocyte apoptosis and extracellular matrix degeneration by restoring the inflammatory microenvironment in OA joints (Zhong et al., 2023). While these studies highlight the advantages of nanoparticles in targeted OA drug delivery, challenges remain, such as competitive binding between nanomaterials and target receptors due to multiple ligands and receptors *in vivo* (Cheng et al., 2023).

For IA management of OA pain and synovitis, hyaluronan-functionalized or receptor-targeted liposomes enable co-delivery of NSAIDs/corticosteroids with extended local exposure. Key risks include burst release and competition with endogenous ligands; design should prioritize release-rate control, ligand density optimization, and reproducible CMC parameters to de-risk translation.

### 3.2 Polymeric nanoparticles

Polymeric nanoparticles have garnered substantial attention in nanomedicine, largely due to what be their adaptable structural properties, relatively straightforward fabrication processes, and what be enhanced stability compared to alternative nanoparticle systems (Pontes et al., 2022). What the evidence tends to suggest, for instance, is that poly (lactic-co-glycolic acid) (PLGA) NPs containing p66shc-siRNA seem to reduce p66shc expression and apparently mitigate pain, cartilage degradation, and inflammatory cytokine release caused by monosodium iodoacetate (MIA) in rat knee joints (Shin et al., 2020). The epidermal growth factor receptor (EGFR), a cell surface receptor, maintain superficial chondrocyte populations, cartilage lubrication, collagen organization, and what might be characterized as cartilage mechanical strength (Jia et al., 2016). What seems particularly noteworthy in this analytical context is the cartilage-targeting strategy utilizing polymeric nanoparticles that was introduced by Wei et al. (2021). Their research suggest that micellar nanoparticles, when combined with a strong EGFR ligand, tend to display what be remarkable stability, minimal toxicity, apparently extended joint retention, and what represent superior cartilage absorption and penetration properties (Wei et al., 2021). What the data suggest is that, through intra-articular administration, these engineered nanoparticles significantly alleviate cartilage degradation, subchondral bone hardening, and pain in a mouse model of osteoarthritis (Wei et al., 2021). Within these evolving conceptual parameters, Maudens et al. investigated a highly kartogenin-loaded nanocrystalline polymeric particle (NPP) system, which generally indicate enhanced chondrogenic and chondroprotective effects (Maudens et al., 2018). Given the complexity of these theoretical relationships, a triamcinolone acetonide extended-release (ER) formulation, authorized in the U.S. for osteoarthritis pain relief, facilitate the gradual release of the corticosteroid from PLGA microspheres into what seems to constitute synovial tissue (Paik et al., 2019). What this approach tends to indicate is an extended therapeutic effect while presumably minimizing systemic adverse reactions (Paik et al., 2019). What emerge from this evidence, collectively, is that these findings seem to point toward the practicality and relative safety of utilizing polymeric nanoparticles in the treatment of osteoarthritis.

PLGA-based carriers are attractive due to established manufacturability and tunable kinetics; however, acidic microclimates during polymer erosion and heterogeneous joint distribution warrant attention. Future studies should report effect sizes with variance, intra-articular residence times, and head-to-head comparisons to standard IA steroids or HA.

Compared with polymeric and inorganic nanoparticles, liposomes offer superior biocompatibility and drug encapsulation efficiency, but they may suffer from rapid clearance and limited long-term stability. In contrast, polymeric nanoparticles often provide longer-term release profiles and higher mechanical stability but may involve more complex manufacturing processes. This comparison underscores the need to select nanoparticles based not only on efficacy but also on translational feasibility and safety.

### 3.3 Inorganic nanoparticles

Typical inorganic nanoparticles—including metals (e.g., silver, iron, gold) and metal oxides—have been widely leveraged across biomedical applications due to their tunable size/shape, high surface area, magnetic/optical responsiveness, and ease of surface functionalization for biocompatibility and targeting (Valot et al., 2021; Sang et al., 2023). In OA, these features translate into opportunities for joint lubrication enhancement, imaging contrast, photothermal or magnetically assisted therapy, and controlled intra-articular drug delivery (Valot et al., 2021; Sang et al., 2023). Alloyed or doped systems (two or more metals) often provide greater structural stability, reduced ion dissolution, and fine-tuned catalytic/photophysical properties compared with single-metal counterparts, which can improve performance under physiological conditions. (Huynh et al., 2020). Given the complexity of these theoretical relationships, Gong et al. developed what constitute a fluorinated graphene (FG) nanosystem with dual functionality—what seems to be long-term lubrication and thermo-responsive drug release—leveraging what the evidence reveal as the ostensibly outstanding near-infrared (NIR) absorption capabilities and what be highly efficient photothermal conversion properties of FG to apparently achieve NIR-triggered drug release (Gong et al., 2023). As proof-of-concept for therapeutic activity, silver nanoparticles have shown anti-osteoarthritic efficacy in murine models—attenuating inflammatory mediators and cartilage degeneration—supporting the potential of inorganic nanomaterials as active agents rather than inert carriers alone (Sang et al., 2023). In parallel, magnetic nanoparticle platforms (e.g., superparamagnetic iron oxide) enable spatially guided, intracartilaginous delivery of chondroprotective molecules and growth-factor mimetics under external magnetic fields, improving retention and penetration within dense cartilage matrices and yielding superior histologic and micro-CT outcomes versus free drug (Jiang et al., 2022). Such magnetically assisted strategies directly address two major hurdles in OA therapy—rapid joint clearance and poor matrix penetration—while maintaining intra-articular localization to limit systemic exposure (Jiang et al., 2022).

Collectively, these advances underscore the promise of engineered inorganic and alloy/doped nanoparticles for OA management. Key next steps include optimizing long-term biocompatibility/biodegradability, minimizing metal-ion–related cytotoxicity, validating safe and efficient on-demand activation (e.g., magnetic or photothermal triggers) *in vivo*, and establishing robust manufacturing/quality frameworks to support clinical translation.

### 3.4 Exosomes

Exosomes are membrane-bound vesicles measuring approximately 30–150 nm in diameter, which encapsulate complex molecular processes occurring in their parent cells (Liu et al., 2020). Through what be the transport of proteins, lipids, mRNAs, miRNAs, lncRNAs, and DNA, exosomes tend to mediate what be critical biological functions including cellular homeostasis maintenance, debris clearance, intercellular and interorgan communication, and targeted molecular delivery (Liang et al., 2021; Krylova and Feng, 2023). He et al. demonstrated that bone marrow mesenchymal stem cell-derived exosomes (BMSC-Exos) significantly upregulated COL2A1 protein expression while downregulating MMP13 in cartilage tissue within a rat OA model (He et al., 2020). These exosomes effectively promoted cartilage repair, enhanced extracellular matrix (ECM) synthesis, and alleviated knee joint pain in OA rats (He et al., 2020). Chen et al. developed an injectable microgel system (CAP/FGF18-hyEXO@HMs) encapsulating hybrid exosomes (CAP/FGF18-hyEXO). By combining *in vivo* FGF18 gene editing with sustained lubrication, this system synergistically enhanced cartilage regeneration, reduced inflammation, and prevented ECM degradation in both *ex vivo* and *in vivo* settings (Chen M. et al., 2024). What appears particularly significant about these findings, within this broader analytical framework, is that with advancing exosome research, their clinical translational potential for OA treatment have become increasingly evident, which suggest what tends to represent a pathway for what might be considered novel therapeutic strategies (Table 1).

**TABLE 1:** Comparative evaluation of nanomaterials for osteoarthritis therapy.

Nanomaterial type	Merits	Limitations	Translational potential
Liposomes	High biocompatibility; versatile drug encapsulation (hydrophilic and hydrophobic); surface easily modifiable	Rapid clearance; limited long-term stability; potential leakage of drugs	Clinically advanced; several formulations tested in trials; suitable for intra-articular delivery
Polymeric Nanoparticles	Sustained and controlled release; mechanical stability; tunable degradation rates	Complex manufacturing; possible immunogenicity; regulatory hurdles	High potential for personalized therapy; some formulations approved for other diseases
Inorganic Nanoparticles	Multifunctional (diagnosis + therapy); strong mechanical and chemical stability; imaging potential	Concerns about long-term biosafety and toxicity; accumulation risk in tissues	Promising for theranostics but limited clinical translation so far
Exosomes	Excellent biological compatibility; natural intercellular communication; intrinsic targeting ability	Scalability and standardization challenges; complex isolation procedures	Emerging as a next-generation therapeutic; high research interest but early-stage clinical use

## 4 Multifunctional nanoparticle strategy

With the advancement of nanotechnology and further research, novel properties and functions of nanomaterials continue to be discovered, enabling scholars to develop diverse therapeutic strategies for OA based on these unique characteristics.

### 4.1 Gene delivery nanoparticles

Gene therapy has emerged as a cutting-edge strategy for addressing osteoarthritis (OA), offering the possibility of modifying disease progression at the molecular level rather than merely alleviating symptoms. Nanoparticles (NPs) provide an effective platform for targeted delivery of therapeutic genes to damaged joint tissues, owing to their tunable physicochemical properties, biocompatibility, and ability to cross biological barriers. For example, Cai et al. designed an innovative nanoparticle system encapsulating plasmid DNA (pDNA) encoding transforming growth factor-β1 (TGF-β1), which was used to modify mesenchymal stem cells (MSCs) (Cai et al., 2023). This strategy enhanced MSC-mediated cartilage repair, as the modified cells exhibited improved proliferation and superior transfection efficiency, leading to significantly greater regeneration in both *ex vivo* and *in vivo* OA models (Cai et al., 2023). Beyond growth factor delivery, gene-loaded nanoparticles have been engineered to regulate critical molecular pathways implicated in OA pathogenesis, including chondrocyte apoptosis, extracellular matrix degradation, and inflammatory cascades. For instance, chitosan–hyaluronic acid hybrid nanoparticles carrying SOX9 or RUNX2 gene constructs have demonstrated the ability to promote chondrogenic differentiation and inhibit hypertrophy of chondrocytes, thereby maintaining cartilage homeostasis (Lu et al., 2014).

Beyond growth factor delivery, recent strategies have targeted both inflammatory signaling and catabolic enzymes via advanced gene editing tools. A notable study by Ponta et al. developed a non-viral CRISPR-Cas9 RNP (ribonucleoprotein) delivery protocol for primary human chondrocytes and other cartilaginous tissues, achieving ∼90% knockout efficiency of RELA (a key NF-κB subunit) and demonstrating reduced inflammatory responses and improved matrix retention under cytokine (e.g., IL-1β) challenge (Ponta et al., 2024). This underscores the potential of gene editing in attenuating the inflammatory cascade in OA.

However, several challenges remain: achieving sustained and spatiotemporally controlled expression of therapeutic genes in the joint environment; minimizing immune responses and off-target effects; improving penetration into dense cartilage matrix; and ensuring translational feasibility (manufacturing, safety, regulatory approval). Future work should aim to develop stimuli-responsive nanoparticles (e.g., responding to pH, oxidative stress, enzymatic activity), combined gene/gene-editing + regenerative strategies (stem cells or scaffold integration), and clinically relevant delivery routes for intraarticular application.

### 4.2 Anti-inflammatory drug-loaded nanoparticles

OA is a chronic disorder involving complex metabolic and inflammatory processes, where inflammation plays a pivotal role in disease progression (Hills, 2000). NPs have been specifically designed for the precise delivery of anti-inflammatory compounds, particularly those targeting critical inflammatory mediators like IL-1β and TNF-α (Chen L. et al., 2024). Research has shown that nanoparticles loaded with IL-1 receptor antagonist (IL-1Ra) effectively reduce synovitis and slow cartilage degradation (Whitmire et al., 2012). In OA, the balance between pro-inflammatory (M1) and anti-inflammatory (M2) macrophage phenotypes within the joint is pivotal (Dai et al., 2018). M2 macrophages, for their anti-inflammatory properties, offer significant therapeutic promise for OA (Dai et al., 2018). Teo et al. engineered gold NPs coated with M2 macrophage membranes (Au-M2 NPs), which effectively reduced MMP13 expression triggered by IL-1β and significantly suppressed nitric oxide release induced by IL-1β, leading to decreased inflammation and reduced matrix degradation (Teo et al., 2022). Yu et al. designed IA-ZIF-8@HMs for OA treatment, which possess both pH-responsive and proton acid-responsive properties, with IA modulating joint inflammation and intracellular oxidative stress (Yu et al., 2023). Jin et al. fabricated an epigallocatechin gallate (EGCG)-loaded hyaluronic acid (HA)/gelatin composite hydrogel, experimentally validated for its effective anti-inflammatory and osteogenic capabilities in a surgically induced OA model (Jin et al., 2020). Additionally, researchers developed PL407-PL338-HA-SFN hydrogels as a drug delivery platform to release sulforaphane (SFN), which downregulates the NF-κB pathway to reduce metalloproteinase expression, thereby treating OA (Monteiro Do Nascimento et al., 2021).

In addition to anti-inflammatory medications, nanoparticles can be engineered to carry a range of therapeutic agents, enabling them to deliver multiple beneficial effects. For example, antibiotic-delivering NPs have been explored. Feng et al. developed PLGA NPs loaded with doxycycline (DOXY) and incorporated them into poly (L-lactic acid) (PLLA) scaffolds, achieving sustained local antibiotic release for prolonged antibacterial efficacy (Feng et al., 2010). Another study demonstrated that injectable polyethylene glycol (PEG) hydrogels delivering lysostaphin ensured efficient localized delivery, effectively treating *Staphylococcus aureus* infections in fractures (Johnson et al., 2018).

### 4.3 ROS-sensitive nanoparticles

Reactive oxygen species (ROS), highly unstable molecules mainly produced by NADPH oxidases and mitochondrial activity, are critically involved in the pathological mechanisms underlying osteoarthritis. Li et al. developed a ROS-responsive functional nanoplatform for OA therapy, comprising borate-stabilized polyphenol-poloxamer assemblies loaded with Dex (Li et al., 2021). This nanomedicine exhibited ROS-triggered drug release kinetics and ROS-scavenging efficacy, effectively suppressing ROS and nitric oxide (NO) production in lipopolysaccharide (LPS)-activated RAW264.7 macrophages, demonstrating therapeutic potential for OA (Li et al., 2021). Lu et al. engineered dual pH- and ROS-responsive nanoparticles loaded with methylprednisolone (MPS) and modified with arginine-glycine-aspartate (RGD) (Lu Y. et al., 2023). These nanoparticles significantly inhibited interleukin-6 (IL-6) and tumor necrosis factor-alpha (TNF-α) expression in the joints of collagen-induced arthritis (CIA) mice, efficiently attenuating joint degradation through the suppression of the NF-κB signaling pathway (Lu Y. et al., 2023). Lv et al. proposed d-RuO_2_ nanospheres exhibiting exceptional nanozyme antioxidant activity. *Ex vivo* experiments confirmed that these nanospheres markedly reduced intracellular ROS levels and downregulated key inflammatory markers, including inducible nitric oxide synthase (iNOS), cyclooxygenase-2 (COX-2), TNF-α, and interleukin-1β (IL-1β) (Lv et al., 2025). *In vivo*, d-RuO_2_ alleviated synovitis, cartilage degeneration, and bone remodeling by inhibiting the ROS/NLRP3/caspase-1 signaling pathway, thereby delaying OA progression (Lv et al., 2025). Yu et al. engineered KGN/Dex-TSPBA@WHMs, a system capable of targeting cartilage specifically, efficiently eliminating ROS, and releasing drugs in response to ROS levels. This innovation shows promising potential for facilitating the regeneration of injured cartilage (Yu et al., 2022). Chondrocyte ferroptosis has emerged as a critical therapeutic target in OA (Cao et al., 2023). Sheng et al. developed fenofibrate (FN)-loaded nanoparticles (FN-CNPs) with ROS-responsive properties. These nanoparticles effectively reduced osteoarthritis progression by regulating ROS levels, enhancing the antioxidant defense system, and improving lipid metabolism in chondrocytes, ultimately preventing ferroptosis in these cells (Sheng et al., 2025).

### 4.4 Stimuli-responsive nanoparticles

Stimuli-responsive nanoparticles release their encapsulated drugs only upon exposure to appropriate triggers or specific conditions. Disease-associated local microenvironmental factors, internal factors like temperature, pH, and oxidative stress, as well as external triggers such as NIR light, offer valuable design principles for creating functionally tailored nanoparticles (Lawson et al., 2021b). For instance, Chen et al. combined a photothermally triggered nitric oxide (NO) nanogenerator with small interfering RNA (siRNA), yielding NO-Hb@siRNA@PLGA-PEG (NHsPP) (Chen et al., 2019). This mechanism efficiently transforms absorbed NIR light into adequate thermal energy, which induces NO production and subsequently inhibits inflammation driven by macrophages (Chen et al., 2019).

Under physiological conditions, the pH of synovial fluid in the knee joint ranges between 7.35 and 7.45 (Xiong et al., 2020). However, in OA, the pH of the joint microenvironment can decrease to below 6.0 (Shirazian et al., 2024). Consequently, pH-sensitive nanoplatforms have been developed for targeted drug delivery in OA treatment. For example, researchers have fabricated pH-responsive PLGA NPs co-encapsulated with ammonium bicarbonate (NH4HCO3), which effectively delay OA progression (Zerrillo et al., 2019). Liu et al. designed a pH-sensitive nanomedicine (TP@NPs) by encapsulating triptolide (TP) into star-shaped amphiphilic block copolymers (POSS-PCL-b-PDMAEMA), demonstrating significant chondroprotective and anti-inflammatory effects (Liu et al., 2021). Jiang et al. developed an acidic environment-responsive hydrogel (NBIF@ZIF-8 MOFs) based on zeolitic imidazolate framework-8 (ZIF-8), enabling stimulus-triggered release of neobavaisoflavone (NBIF). This system synergistically modulates immune responses and promotes cartilage defect repair through the combined action of NBIF and the hydrogel matrix (Jiang et al., 2023).

### 4.5 Cartilage repair and regeneration scaffolds

Biomaterial scaffolds incorporating nanotechnology have become indispensable in cartilage tissue engineering, as they provide a three-dimensional (3D) extracellular matrix–mimicking architecture that not only facilitates cell adhesion, proliferation, and differentiation but also enables precise spatial and temporal delivery of bioactive molecules. Such scaffolds can be engineered to release growth-promoting and anti-inflammatory agents in a controlled manner, thereby fostering cartilage repair while simultaneously supporting subchondral bone regeneration.

Consistent with the ‘scaffold × gene therapy’ strategy discussed above, Venkatesan et al., used an alginate hydrogel to guide rAAV-mediated delivery of FGF-2/TGF-β, significantly enhancing extracellular matrix synthesis by human meniscal fibrochondrocytes and promoting tissue repair (Venkatesan et al., 2024). In a related study, Zhang et al. explored the use of a pH-sensitive metal-organic framework (MOF), MIL-101-NH2, to simultaneously deliver the anti-inflammatory agent curcumin (CCM) and siRNA designed to inhibit hypoxia-inducible factor-2α (HIF-2) (Zhang Z.-J. et al., 2023). By silencing the HIF-2α gene and suppressing inflammatory responses and cartilage degeneration in OA, this MOF demonstrated promising therapeutic potential for OA treatment (Zhang Z.-J. et al., 2023). Shin et al. designed a novel mesenchymal stem cell (MSC) platform, named Edu-MSCs-AuS-TA, where gold nanoparticles (AuNPs) loaded with triamcinolone acetonide (TA) were attached to the MSC surface to amplify their anti-inflammatory properties (Shin et al., 2024). By combining AuNPs with MSCs and utilizing near-infrared laser-assisted photothermal therapy (PTT), this approach promoted anti-inflammatory effects and induced macrophage repolarization (Shin et al., 2024). Notably, it significantly alleviated arthritis-associated pain, improved overall motor function, and even induced cartilage regeneration in advanced-stage arthritis models (Shin et al., 2024). Recent advances further underscore the versatility of nanostructured scaffolds: hydrogel–nanoparticle composites can provide mechanical reinforcement, lubricating function, and ROS/pH-responsiveness, thus allowing drug release tailored to the inflamed OA microenvironment (Yu et al., 2022). Moreover, exosome-functionalized nanoscaffolds have been shown to promote chondrocyte proliferation and inhibit apoptosis, offering a synergistic strategy that couples regenerative cell therapy with nanomaterial-based delivery platforms (Chen M. et al., 2024). In parallel, recent studies in chronic inflammatory wound models have demonstrated the broad versatility of nanostructured hydrogels. For example, Gong et al. engineered a metal–polyphenol nanocomposite hydrogel capable of reprogramming the metabolic microenvironment and promoting robust angiogenesis in diabetic foot ulcers (Gong et al., 2025). Similarly, Li et al. reported a smart hydrogel dressing with dual-barrier drug delivery properties that enhanced healing of chronic infectious wounds (Li Y. et al., 2024). Moreover, He et al. developed a cascade-nanozyme-loaded hydrogel that facilitated revascularization and modulated macrophage phenotypes, thereby accelerating tissue regeneration (He et al., 2025). These findings, although outside the OA field, provide translational insights for designing multifunctional hydrogel scaffolds to improve cartilage repair and microenvironmental regulation in osteoarthritis.

Collectively, these findings highlight that scaffold-based nanotechnologies represent a convergence point of material science, molecular therapy, and regenerative medicine, and they are likely to play a pivotal role in the next-generation of clinically translatable OA treatments.

### 4.6 Lubricating functional nanoparticles

NPs exhibit unique mechanical and tribological properties, enabling them to enhance lubrication by modulating interfacial friction, infiltrating narrow gaps to form protective layers, and functioning as intermediate ball bearings between surfaces (Altman et al., 2015; Lawson et al., 2021b). Hyaluronic acid (HA), a key component of cartilage matrix and synovial fluid, possesses excellent viscoelasticity and strain-dependent properties, contributing to its distinctive hydrodynamic behavior and effective joint lubrication (Lu K.-H. et al., 2023). For decades, intra-articular injections of HA-based viscosupplements have been a widely adopted approach for managing osteoarthritis (OA)-related complications (Gonzales et al., 2023). The evidence-based clinical practice guidelines for knee OA, updated in both 2019 and 2023, endorse the use of hyaluronic acid (HA)-based viscosupplementation as a therapeutic approach (Barthold et al., 2024). Zheng et al. developed HA-MPC nanospheres, which demonstrated a 40% reduction in the coefficient of friction compared to HA alone (Zheng et al., 2022). These nanospheres can be administered via intra-articular injection to effectively treat OA by restoring joint lubrication (Zheng et al., 2022).

### 4.7 Nanoparticles for imaging diagnosis

In clinical practice, imaging modalities such as computed tomography (CT), magnetic resonance imaging (MRI), and photoacoustic imaging (PAI) be typically employed to assess what constitute cartilage degeneration in OA patients (Braun and Gold, 2012; Lawson et al., 2021a). With continuous innovations in nanotechnology, researchers have explored NPs with unique physical properties to develop novel contrast agents that prolong *in vivo* retention, enhance sensitivity, reduce toxicity, and enable early OA diagnosis and progression monitoring (Mohammadinejad et al., 2020; Zhang et al., 2025). Zhang et al. designed a novel contrast agent platform utilizing cationic nanoparticles (NPs), which integrates cationic peptide carriers (CPCs) with multivalent poly (ethylene glycol)-modified avidin (mAv) NPs (Zhang C. et al., 2023). When combined with ioxaglate (IOX), this system effectively penetrates cartilage tissue via electrostatic interactions, significantly enhancing CT imaging (Zhang C. et al., 2023). In contrast to traditional IOX, this approach produces comparable CT signals at doses approximately 40 times lower, significantly minimizing toxicity concerns while offering a highly sensitive and safe method for early OA diagnosis and progression (Zhang C. et al., 2023; Table 2). In a separate study, Wu et al. engineered ultrasmall superparamagnetic iron oxide nanoparticles (SPIONs) modified with the WYRGRL peptide ligand, designed to specifically target type II collagen within the cartilage matrix (Wu et al., 2023). As type II collagen degrades during OA progression, distinct MRI signal patterns emerge in OA subjects versus controls (Wu et al., 2023). This study introduces a promising approach for transporting nanoscale imaging agents to articular cartilage, which could facilitate the diagnosis of OA progression (Wu et al., 2023). In addition, a recent review summarized the applications of SPIONs in the diagnosis and treatment of bone and joint diseases, highlighting mechanisms, imaging sensitivity, biodistribution, and safety considerations (Zhu et al., 2024). PAI has emerged as a noninvasive imaging modality for OA diagnosis and monitoring (Park et al., 2024). Shen et al. engineered a photoacoustic probe (Au@PDA-WL NPs) designed to target type II collagen, consisting of gold NPs encapsulated in polydopamine (PDA) and functionalized with the WYRGRL peptide (Shen et al., 2023). Within this broader analytical framework, notably about these findings is that this innovation tends to suggest what be enhanced identification of early osteoarthritic alterations while seemingly creating novel opportunities for disease tracking and therapeutic investigations (Shen et al., 2023).

**TABLE 2:** Toxicity profiles of selected inorganic nanoparticles used in OA models.

Nanoparticle	Dosage range	Model system	Route of administration	Toxicological effects
TiO_2_	10–100 μg/mL	Chondrocytes (*in vitro*)	Direct culture	ROS generation at >50 μg/mL
CuO	5–50 mg/kg	Rat (*in vivo*)	Intra-articular injection	Dose-dependent inflammation
CeO_2_	10–200 μg/mL	RAW264.7 macrophages	*In vitro*	Low ROS and good biocompatibility
Mg(OH)_2_	1–10 mM	Human OA chondrocytes	*In vitro*	Safe below 5 mM; cytotoxic at higher concentrations

What the evidence reveal, considering the nuanced nature of these findings, is that these studies provide evidence that may support the multifaceted potential of NPs in OA diagnosis and treatment, expanding therapeutic options and what offer new hope for improving patient outcomes in the majority of cases.

## 5 Challenges and future directions

With the increasing aging and obese populations, the occurrence rate of OA is expected to rise progressively. At present, the majority of clinical approaches for managing OA aim to slow disease advancement, reduce pain, and enhance mobility. Nevertheless, the intricate molecular and cellular changes occurring in the cartilage of OA patients pose significant challenges, optimal therapeutic outcomes are difficult to achieve through conventional clinical approaches. Therefore, developing a treatment plan that enables long-term disease management and improves patient prognosis remains an urgent priority.

Progress in nanotechnology, combined with an enhanced comprehension of OA pathophysiology, has facilitated the application of nanoparticles’ distinctive characteristics. These advancements allow for sustained drug delivery, increased intra-articular drug persistence, and optimized therapeutic outcomes via functional modification approaches. These systems can also reduce therapeutic dosages, decrease administration frequency, enhance pharmacological effects, and minimize off-target toxicity. Various nanoparticles have been extensively explored for OA and other diseases, including chondroprotective therapies (Yang et al., 2020)and nanomaterial-based scaffolds for osteogenesis (Mahboudi et al., 2018). As a result, nanotechnology has become a highly promising field for creating novel therapeutic approaches to address OA.

Nevertheless, several technical challenges and limitations must be overcome before nanoparticles can be widely adopted for OA therapy. Liposomes are clinically well-studied but suffer from short circulation times. Polymeric nanoparticles provide superior sustained release but raise manufacturing and regulatory complexity. Inorganic nanoparticles offer multifunctional diagnostic and therapeutic capabilities, yet their long-term biosafety remains uncertain. Exosomes present excellent biological compatibility but face scalability and standardization challenges. These comparisons highlight that no single platform is universally optimal, and the maturity of each approach varies considerably. Key constraints include the scarcity of *in vivo* studies and the high production costs of NPs (Grayson and Brown, 2025). Additionally, synthetic nanomaterials are susceptible to the complex *in vivo* microenvironment, often failing to achieve their intended functionality, biocompatibility, and other design objectives (Farjadian et al., 2019). Furthermore, for nanoparticles to become a mainstream clinical treatment for OA, standardized evaluation systems for nanomedicines, clinical treatment guidelines, and regulatory frameworks must be established. Achieving these goals will require extensive, reliable data on safety and efficacy.

In summary, nanomaterials exhibit significant potential in the diagnosis and treatment of OA. They can be utilized not only for early OA diagnosis and monitoring disease progression but also for enhancing therapeutic efficacy and improving patients’ quality of life by targeting cartilage and synovium. Looking ahead, we anticipate that future investigations will increasingly focus on: (1) intelligent and multi-responsive drug delivery systems capable of dynamic, on-demand release under specific pathological stimuli; (2) precision and personalized nanomedicine tailored to individual OA phenotypes and patient-specific joint microenvironments; (3) biomimetic nanomaterials that integrate with biological tissues to improve safety and long-term biocompatibility; and (4) interdisciplinary strategies that combine nanotechnology with gene therapy, stem cell therapy, and regenerative medicine. Importantly, the establishment of standardized regulatory guidelines and robust evaluation criteria for nanomedicines will be crucial to facilitate clinical translation. With these directions, nanotechnology is expected to reshape OA management by enabling more precise, safer, and effective treatment paradigms”.
